# Childhood Tuberculosis in Northern Viet Nam: A Review of 103 Cases

**DOI:** 10.1371/journal.pone.0097267

**Published:** 2014-05-12

**Authors:** Robert J. Blount, Bao Tran, Leah G. Jarlsberg, Ha Phan, Van Thanh Hoang, Nhung Viet Nguyen, Deborah A. Lewinsohn, Payam Nahid

**Affiliations:** 1 Division of Pulmonary and Critical Care Medicine, University of California San Francisco, San Francisco, California, United States of America; 2 National Lung Hospital, Ha Noi, Viet Nam; 3 Viet Nam National Tuberculosis Program, Ha Noi, Viet Nam; 4 Department of Pediatrics, Oregon Health & Science University, Portland, Oregon, United States of America; INIAV, I.P.- National Institute of Agriculture and Veterinary Research, Portugal

## Abstract

**Background:**

Childhood tuberculosis causes significant morbidity and mortality in Southeast Asia, yet little is known about the epidemiology and clinical characteristics of this disease in Viet Nam.

**Objectives:**

To determine the demographics, clinical presentations, radiographic and microbiologic findings, treatment regimens, and outcomes of children admitted with tuberculosis (TB) to a national referral hospital in Viet Nam.

**Methods:**

We conducted a retrospective case series study of children ≤ 15 years old with bacteriologically confirmed or clinically diagnosed TB admitted to a national referral hospital in Ha Noi, Viet Nam from January through December 2007.

**Results:**

One hundred three children were identified: median age 5 years (IQR 2-10), 44% female, 99% Kinh ethnicity, 27% residing in Ha Noi, 88% with BCG vaccination, 27% with known TB contact, and 38% malnourished. Intrathoracic TB was present in 62%, extrathoracic in 52%, both intra and extrathoracic in 19%, and undetermined site in 5%. The most common extrathoracic manifestation was peripheral lymphadenitis, and children under 5 were more likely to have miliary TB or both intra and extrathoracic TB. Fever and failure to thrive were common presenting symptoms among all participants (65% and 56%, respectively), 66% of those with intrathoracic TB presented with cough, and 92% of those with TB meningitis presented with severe neurologic impairment. Acid-fast bacilli smears and mycobacterial cultures were positive in 18% and 21% of children tested, and histopathology was positive in 88% of those biopsied. There were no adverse drug reactions necessitating change in therapy, and no inpatient mortality.

**Conclusions:**

Extrathoracic TB was common, treatment well tolerated and clinical outcomes excellent. Culture confirmation rates were low and emphasize the need for improved diagnostics.

## Introduction

Of the 8.6 million people diagnosed with tuberculosis (TB) disease in 2012, an estimated 530, 000 (6%) were children under 15 years old [1]. The actual percentage of TB occurring in children is likely as high as 11–15%, given the lower case ascertainment rates in children compared with adults [2], [3]. The impact of TB is particularly profound in young children because they progress more rapidly to TB disease and are more susceptible to severe TB [4]. Viet Nam, one of 22 high burden countries that collectively account for about 80% of the world's TB cases, has an estimated TB disease incidence of 147 cases per 100,000 [1], with an estimated prevalence of TB infection (as measured by Tuberculin Skin Test (TST) ≥ 10 mm) of 16.7% among 6–14 year-olds [5]. Despite the suspected high burden of TB disease among children in Viet Nam, there have been few childhood TB studies in this region, and the clinical manifestations and treatment outcomes of childhood TB in Viet Nam remain unclear. Bacteriologic confirmation of childhood TB is low [2], [6]–[12], necessitating a heavy reliance on clinical characteristics to direct diagnosis and treatment in the majority of cases. As such, a better understanding of the complex and heterogeneous clinical manifestations of childhood TB is necessary for developing and implementing more effective TB prevention, diagnostic, and treatment strategies [13]. To address this need, we conducted a case series study at a large referral hospital in Northern Viet Nam to better characterize the demographics, clinical presentation, radiographic and microbiologic findings, treatment, and clinical outcomes of children diagnosed with TB disease in Northern Viet Nam.

## Methods

We conducted a hospital based retrospective case series study of children ≤ 15 years old with TB disease hospitalized at the National Lung Hospital (NLH) in Ha Noi, Viet Nam between January 1 and December 31, 2007. NLH is Northern Viet Nam's only nationally designated referral hospital for childhood TB, and is headquarters for the Viet Nam National Tuberculosis Control Program. Children with suspected TB were referred to the NLH by several mechanisms: (1) self/family referral through the emergency department, (2) from district or provincial hospitals throughout Northern Viet Nam, (3) and from the National Hospital of Pediatrics in Ha Noi. Two physicians with extensive clinical TB experience, independent of the treating physicians, reviewed each participant's demographics, medical history, presenting symptoms, physical exam, laboratory results, chest radiograph (CXR) findings, and microbiologic and histopathologic results; and collected this data using a standardized chart abstraction form (see Form S1). They abstracted general symptoms for all subjects, and site specific symptoms based on the involved site of TB infection. In this study “TB Contact” was defined as exposure within three months of symptom onset to a person with TB disease, as ascertained through self/family report and not through formal household contact investigations. All children ≤ 15 years old admitted to NLH with TB in 2007 were eligible for inclusion in the study, and those with incomplete or missing charts were excluded.

We analyzed nutritional status for each participant ≤ 10 years old by calculating weight-for-age z-scores based on the World Health Organization (WHO) Multicentre Growth Reference Study [14] using WHO Anthro and Anthro Plus software version 3.2.2, 2011. Weight-for-age z-score reference data was not available for those > 10 years old. We used WHO definitions for malnutrition: no malnutrition, z-score between −2 and 2; moderate malnutrition, z-score<−2 to −3; and severe malnutrition, < −3. Failure to thrive was defined as downward deviation from the expected WHO weight-for-age growth curve trajectory or a z-score < −2.

The WHO diagnostic approach for evaluating children suspected of having TB [15] was employed: after clinical history was obtained and physical exam performed, up to three specimens were collected from each child and submitted for acid-fast bacilli (AFB) examination (Ziehl Neelsen) and for mycobacterial culture on solid Lowenstein-Jensen media or liquid media (Middlebrook 7H9 broth, Mycobacteria Growth Indicator Tubes (BACTEC MGIT), Becton Dickinson, Forest Lake, NJ). Drug susceptibility to isoniazid and rifampicin was performed, with additional susceptibility testing to pyrazinamide, ethambutol, and streptomycin for specimens resistant to rifampin or isoniazid. Gastric lavage was performed for children unable to expectorate sputum, and for children with extrathoracic manifestations, additional specimens were obtained from involved sites by lumbar puncture, thoracentesis, paracentesis, fine-needle aspiration, or tissue biopsy. In the absence of bacteriologic confirmation, diagnosis was based on radiographic, histopathologic, and clinical data including exposure to household members with pulmonary TB, presenting symptoms, inadequate response to routine empiric antibiotics, a positive TST (induration of ≥10 mm in HIV-uninfected and ≥5 mm in HIV-infected children), and response to antituberculosis therapy. Severe TB was defined as miliary and/or meningeal TB. CXRs were obtained on all children and read by NLH staff radiologists trained in pediatric TB imaging. Radiologists were not blinded to clinical information. All pathology samples were reviewed by an NLH pathologist. HIV testing was requested at the discretion of the attending physician. HIV was confirmed using two rapid enzyme immunoassays: SD Bioline HIV 1/2 (Standard Diagnostics, Kyonggi-do, South Korea) and Determine (Abbott Laboratories, Abbot Park, IL, USA), with a third immunoassay testing those with discordant results (Genscreen HIV 1/2, Bio-Rad Laboratories, Paris, France). Those with at least two positive tests were diagnosed with HIV.

Treatment regimens were determined by WHO childhood TB treatment guidelines [16], with the important exception that streptomycin was given instead of ethambutol (E) during intensive phase treatment. Children with HIV, new pulmonary TB (smear-positive or smear-negative with extensive parenchymal involvement), or severe extrapulmonary TB were prescribed 2 months of intensive phase treatment with rifampin (R), isoniazid (H), pyrazinamide (Z), and streptomycin (S) (2RHZS) followed by 4 months of continuation phase treatment with rifampin and isoniazid (4RH) while children with new smear-negative pulmonary TB or mild extrapulmonary TB were prescribed 2 months of isoniazid, rifampin, and pyrazinamide (2RHZ) followed by 4RH. Children with previously treated smear-positive pulmonary TB were retreated with 2RHZES, followed by 1RHZE, followed by 5RHE. Intensive phase treatment was uniformly initiated in the inpatient setting. Hepatotoxicity while on antituberculosis medication was recognized by jaundice, and biochemical testing was not routinely performed to screen for hepatotoxicity. The NLH's standard practices were to discharge patients to home once clinically stable and tolerating oral antituberculosis medications, with planned follow-up at NLH or local health centers 2 weeks after discharge, at the completion of intensive phase treatment, and every 2 months thereafter until treatment completion. Children with clinical deterioration necessitating intensive care management were transferred to the National Hospital of Pediatrics Intensive Care Unit in Ha Noi.

The study was approved by the institutional review boards at the University of California, San Francisco and the National Lung Hospital, Ha Noi. Written consent was not obtained for this specific retrospective chart abstraction study. All patient data was de-identified using the following methods. Each patient was assigned a study identification number, and only this number was used to identify each subject in data bases and data analysis, with the file linking study identification numbers to personal identifying information (name, address, and medical record number) kept separately and not available to the data analysis team.

We assessed differences in demographics, clinical characteristics, and microbiologic results by age category in unadjusted analyses using the Chi-squared test for categorical data (Fisher exact where appropriate) and Wilcoxon rank sum test for continuous data. We assessed predictors of clinical presentation, site of TB infection, TST positivity, and clinical outcomes using multivariable logistic regression analyses, including potential confounders based on prior literature, biologic plausibility, and p<0.20 in bivariate analyses. Statistical analyses were performed using Stata 13SE (Stata, Inc., College Station, TX).

## Results

### Demographics

**Figure 1:**
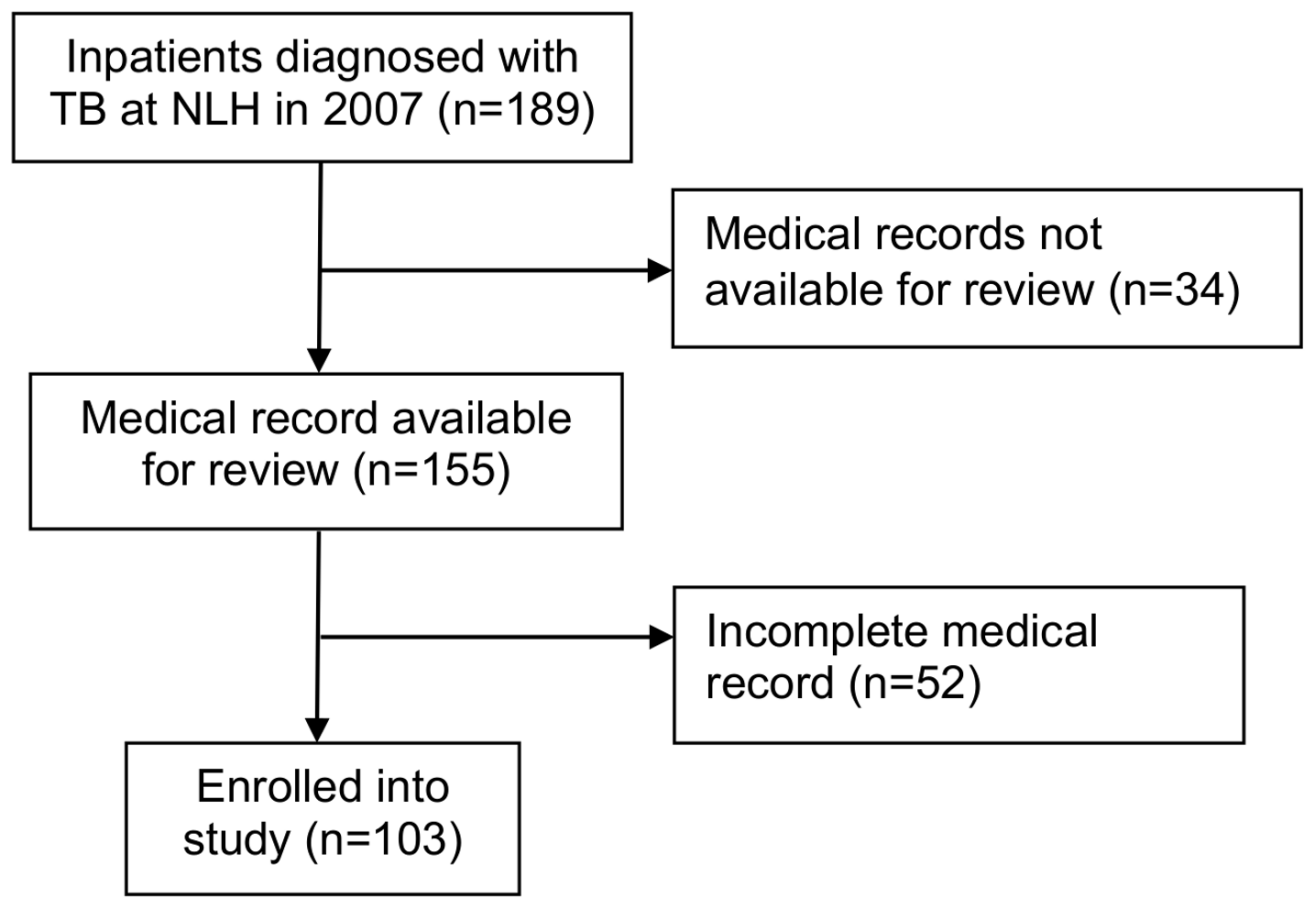
Study enrollment.

A final diagnosis of TB disease was determined for 189 children ≤15 years old admitted to NLH in 2007. Of these, complete medical records were available for 103 (Figure 1). The median age of participants was 5 years (IQR 2-10 years, range 2 months - 15 years old). Twenty-one (20%) were less than 2 years old and nearly half (n = 48, 47%) were < 5 years old (Table 1). Ethnicity and nationality were available for 101 participants. Ninety nine percent were of Kinh ethnicity (compared with 85.7% Kinh by nationwide 2009 census) [17] and all reported Vietnamese citizenship. Twenty-eight (27%) participants resided in Ha Noi, 76 (74%) within 110km of the hospital, and all but one (n = 102, 99%) resided in Northern Viet Nam. Participants were referred from one of three sources: self/family referred through the emergency department (n = 16, 16%), district or provincial hospitals (n = 41, 40%), or the National Hospital of Pediatrics (n = 45, 44%).

**Table 1:** Demographics, History, and Clinical Characteristics of Participants According to Age (n = 103*).

Characteristic	All children	0–4 years	5–15 years	P value
	n (%)	n (%)	n (%)	
Gender	n = 103	n = 48	n = 55	
Male	58 (56)	24 (50)	34 (62)	0.23
Female	45 (44)	24 (50)	21 (38)	
Ethnicity	n = 101	n = 47	n = 54	
Kinh	100 (99)	47 (100)	53 (98)	1.0
Other	1 (1)	0	1 (2)	
Nationality	n = 101	n = 47	n = 54	
Vietnamese	101 (100)	47 (100)	54 (100)	1.0
Other	0	0	0	
Residence	n = 103	n = 48	n = 55	
Hanoi city	28 (27)	12 (25)	16 (29)	0.64
Northern Vietnam	102 (99)	47 (98)	55 (100)	0.47
Southern Vietnam	1 (1)	1 (2)	0	
Referred from:	n = 102	n = 47	n = 55	
Self/family referral	16 (16)	5 (11)	11 (20)	0.48
District or provincial hospital	41 (40)	20 (43)	21 (38)	
National Hospital of Pediatrics	45 (44)	22 (47)	23 (42)	
Received BCG vaccine	91 (88)	40 (83)	51 (93)	0.14
History of prior TB	14 (14)	8 (17)	6 (11)	0.57
TST Results†	n = 99	n = 46	n = 53	
Positive	50 (51)	21 (46)	29 (55)	0.37
Negative	49 (49)	25 (54)	24 (45)	
Induration of positive TST; mean (± SD in mm) (n = 50)	16 (3.2)	15 (2.8)	17 (3.3)	0.15
Reported TB contact	28 (27)	13 (27)	15 (27)	0.98
HIV status	n = 103	n = 48	n = 55	
Positive	9 (9)	9 (19)	0	0.001
Negative	18 (17)	10 (21)	8 (15)	
Unknown	76 (74)	29 (60)	47(85)	
Nutritional status	n = 76	n = 45	n = 31‡	
Normal	47 (62)	29 (64)	18 (58)	0.84
Moderate malnutrition	13 (17)	7 (16)	6 (19)	
Severe malnutrition	16 (21)	9 (20)	7 (23)	
Weight-for-age z score; mean (± SD) (n = 76)	−1.58 (1.48)	−1.65 (1.46)	−1.49 (1.54)‡	0.74

Definition of abbreviations: BCG  =  Bacillus Calmette-Guerin; TST  =  tuberculin skin test; SD  =  one standard deviation.

*n = 103 for all children, 48 for 0–4 year-olds, and 55 for 5–15 year-olds, except where indicated.

† A positive TST was defined as ≥5mm if participant HIV-infected, otherwise ≥10mm.

‡ WHO z-scores not available for 11–15 year-olds.

### Clinical characteristics

Most children were BCG (Bacillus Calmette-Guerin) vaccinated (n = 91, 88%) (Table 1). TST was positive in 51% and a source case identified in 27% of children diagnosed with TB. HIV testing was positive in 9% of cases, but only 27 were tested. Thirty eight percent of children were either moderately or severely malnourished, and poor nutritional status was associated with increased extrathoracic TB and decreased TST positivity. For every unit decrease in z-score, there was a 1.5 fold higher odds of having extrathoracic TB (OR 1.5, 95%CI 1.04–2.0, p = 0.03) and a non-statistically significant 1.3 fold higher odds of having a negative TST (OR 1.3, 95%CI 0.96–1.9, p = 0.09).

Intrathoracic TB was diagnosed in 64 (62%) patients, extrathoracic TB in 54 (52%), both intra and extrathoracic TB in 20 (19%), and undetermined site in 5 (5%) (Table 2). Of those with extrathoracic TB, the most common sites were extrathoracic lymphatic (n = 23), osteoarticular (n = 12), meningeal (n = 12), and miliary (n = 7) disease. Children 0–4 years old were more likely to have both intra and extrathoracic TB (p = 0.001), miliary TB (p = 0.048), or TB of undetermined site (p = 0.02) than 5–15 year-olds. Seventeen children (17%) had severe TB, and a lower proportion of BCG vaccinated children had severe TB (13 out of 91 (14%)) compared with BCG unvaccinated children (4 out of 12 (33%)) (p = 0.11). Using a cutoff of 3 years of age, severe TB was more common in children under 3 (8/28 (29%)) compared with children age 3–15 (9/75 (12%)) (p = 0.044). Most patients presented with fever (n = 67, 65%) or failure to thrive (n = 58, 56%) (Table 3). Intrathoracic TB and severe TB were each independently associated with increased odds of fever compared with those with non-severe extrathoracic TB (OR 8.57, 95%CI 2.97–24.7, p<0.001 and OR 14.0, 95%CI 1.42–138, p = 0.02, respectively). Among those with intrathoracic TB, cough was also common (66%) while only 4 experienced hemoptysis (6%) (3 of these had cavitary TB). Chest pain was uncommon (16%), identified only in children 5–15 years old. Predominant abnormalities found on CXR included lobar consolidation (41%), nodular lesions (27%), pleural effusions (22%), and intrathoracic adenopathy (19%) (Table 4). Pleural effusions were more common in 5–15 year-olds (39%) than 0–4 year-olds (6%) (p = 0.02). The clinical triad of recent TB contact, positive TST, and abnormal CXR findings was evident in only 14 participants with intrathoracic TB (22%).

**Table 2:** Site of TB Disease According to Age.

	All children	0–4 years	5–15 years	P value
	n (%)*	n (%)	n (%)	
Site of TB infection	n = 103	n = 48	n = 55	
Intrathoracic	64 (62)	33 (69)	31 (48)	0.20
Extrathoracic	54 (52)	26 (54)	28 (51)	0.74
Both intra and extrathoracic	20 (19)	16 (33)	4 (7)	0.001
Extrathoracic lymphatic	23 (22)	12 (25)	11 (20)	0.54
Osteoarticular	12 (12)	4 (8)	8 (15)	0.37
Meningeal	12 (12)	6 (13)	6 (11)	1.0
Miliary	7 (7)	6 (13)	1 (2)	0.048
Abdominal	4 (4)	1 (2)	3 (5)	0.62
Other	2 (2)	2 (4)	0	0.22
Undetermined site	5 (5)	5 (10)	0	0.02
Severe TB†	17 (17)	10 (21)	7 (13)	0.27

*Column percentages add up to > 100% because many children had multiple sites of infection.

† Severe TB is defined as presence of meningeal and/or miliary TB.

**Table 3:** Presenting Symptoms According to Site of Disease and Age.

	All children	0–4 years	5–15 years	P value
Symptom or Finding	n (%)	n (%)	n (%)	
General symptoms, all TB	n = 103	n = 48	n = 55	
Fever	67 (65)	34 (71)	33 (60)	0.25
Failure to thrive	58 (56)	31 (65)	27 (49)	0.11
Intrathoracic TB symptoms	n = 64	n = 33	n = 31	
Fever	52 (81)	29 (88)	23 (74)	0.21
Failure to thrive	37 (58)	21 (64)	16 (51)	0.33
Cough	42 (66)	21 (64)	21 (68)	0.77
Dyspnea	11 (17)	4 (12)	7 (23)	0.39
Chest Pain	10 (16)	0	10 (32)	0.001
Hemoptysis	4 (6)	2 (6)	2 (6)	0.85
Extrathoracic TB symptoms	n = 54	n = 26	n = 28	
Fever	30 (56)	18 (69)	12 (43)	0.05
Failure to thrive	31 (57)	17 (65)	14 (50)	0.25
TB meningitis symptoms	n = 12	n = 6	n = 6	
Fever	10 (83)	5 (83)	5 (83)	1.0
Failure to thrive	5 (42)	3 (50)	2 (33)	1.0
Coma	7 (58)	3 (50)	4 (67)	1.0
Seizures	5 (42)	3 (50)	2 (33)	1.0
Limb paresis	4 (33)	2 (33)	2 (33)	1.0
Cranial nerve palsy	1 (8)	1 (17)	0	1.0
Neck pain or stiffness	6 (50)	1 (17)	5 (83)	0.08
Headache	5 (42)	0	5 (83)	0.02
Miliary TB	n = 7	n = 6	n = 1	
Fever	6 (86)	6 (100)	0	0.14
Failure to thrive	5 (71)	4 (67)	1 (100)	1.0

**Table 4:** CXR Findings in Those with Intrathoracic TB According to Age.

	All children	0–4 years	5–15 years	P value
	n (%)	n (%)	n (%)	
CXR findings	n = 64	n = 33	n = 31	
Lobar consolidation	26 (41)	14 (42)	12 (32)	0.76
Nodular lesions	17 (27)	12 (36)	5 (16)	0.09
Pleural effusion	14 (22)	2 (6)	12 (39)	0.02
Intrathoracic adenopathy	12 (19)	7 (21)	5 (16)	0.75
Pleural thickening	7 (11)	1 (3)	6 (19)	0.050
Miliary	7 (11)	6 (18)	1 (3)	0.11
Cavitation	6 (9)	3 (9)	3 (10)	1.0
Atelectasis	2 (3)	1 (3)	1 (3)	1.0
Bronchial thickening	1 (2)	1 (3)	0	1.0

Extrathoracic TB symptoms and physical exam findings were site specific. Among those with osteoarticular TB (6 with spondylitis and 6 with arthritis), 11 experienced localized pain at the affected site (92%) and all with spondylitis had a gibbus deformity on exam. Eleven (92%) of those with TB meningitis (n = 12) experienced at least one symptom or sign reflective of severe disease: coma (58%), seizures (42%), limb paresis (33%), or cranial nerve palsy (8%) (Table 3). The most common symptoms in participants with miliary TB (n = 7) were fever (n = 6, 86%) and failure to thrive (n = 5, 71%). Of the 4 participants with abdominal TB, 3 experienced abdominal pain and/or distention.

### Microbiology and histopathology

Fourteen of 76 children (18%) with at least one smear performed were smear positive, and 15 of 72 children (21%) with at least one culture performed were Mtb culture positive (Table 5). Thirty of the 34 participants biopsied (88%) had caseating granulomas consistent with TB on histopathologic examination. Among those who were cultured, infants were most likely to have a positive culture (89% compared with 13% for 1–4 year-olds and 10% for 5–15 year olds (p<0.001), and those with both intra and extrathoracic disease were more likely to have a positive culture (n = 9/17, 53%) than children with intrathoracic disease alone (n = 6/34, 18%) or extrathoracic disease alone (n = 0/16, 0%) (p = 0.001). Smears were performed on a total of 119 specimens from the following sources: gastric lavage (n = 49, 41%), expectorated sputum (n = 17, 14%), bronchoalveolar lavage (n = 19, 16%), cerebral spinal fluid (n = 15, 13%), pleural fluid (n = 11, 9%), and other sources – feces, peritoneal, urine, testicular, abscess (n = 8, 7%). Smears were positive in 18 specimens tested (15%). Among smear positive specimens, culture data was available for 16. Cultures were positive in 12 (75%). Among the 4 specimens that were smear positive but culture negative 2 were fecal specimens, 1 was expectorated sputum, and 1 was gastric lavage. Drug susceptibility was performed on 14 specimens: 13 were pan susceptible, while one demonstrated resistance to isoniazid.

**Table 5:** Microbiologic and Histopathologic Results According to Site of TB Infection and Age.

Test according to TB site	Positive test, n (%)				
	All children	<12 months	1–4 years	5–15 years	P value
Any Site	n = 103	n = 9	n = 39	n = 55	
Smear	14/76 (18)	4/9 (44)	6/33 (18)	4/34 (12)	0.09
Culture	15/72* (21)	8/9 (89)	4/32 (13)	3/31 (10)	<0.001
Histopathology	30/34 (88)	2/4 (50)	15/15 (100)	13/15 (87)	0.03
Intrathoracic TB only	n = 44	n = 3	n = 14	n = 27	
Smear	8/37 (22)	3/3 (100)	1/14 (7)	4/20 (20)	0.003
Culture	6/34 (18)	3/3 (100)	0/13 (0)	3/18 (17)	0.001
Histopathology	4/5 (80)	0/1 (0)	4/4 (100)	0	0.2
Extrathoracic TB only	n = 34	n = 0	n = 10	n = 24	
Smear	1/17 (6)	0	1/6 (17)	0/11 (0)	0.35
Culture	0/16 (0)	0	0/6 (0)	0/10 (0)	
Histopathology	15/17 (88)	0	5/5 (100)	10/12 (83)	1.0
Both Intra and Extrathoracic TB	n = 20	n = 5	n = 11	n = 4	
Smear	5/17 (29)	1/5 (20)	4/9 (44)	0/3 (0)	0.49
Culture	9/17 (53)	5/5 (100)	4/9 (44)	0/3 (0)	0.02
Histopathology	11/12 (92)	2/3 (67)	6/6 (100)	3/3 (100)	0.5
Unknown Site	n = 5	n = 1	n = 4	n = 0	
Smear	0/5	0/1 (0)	0/4 (0)	0	
Culture	0/5	0/1 (0)	0/4 (0)	0	
Histopathology	0	0	0	0	

*Missing data on 4 specimen cultures.

### Treatment regimens and outcome

The median length of hospital stay was 36 days (IQR 21–60 days). Most participants were prescribed intensive phase treatment with either 2RHZS (n = 68, 66%) or 2RHZ (n = 28, 27%), followed by continuation phase treatment with 4RH (n = 101, 98%). All but one prescribed 2RHZS (n = 67, 99%) were diagnosed with new smear-positive pulmonary TB, smear-negative pulmonary TB with extensive parenchymal involvement, or extrapulmonary TB, and 26 (93%) of those prescribed 2RHZ were diagnosed with either new smear-negative pulmonary TB or non-severe extrapulmonary TB. Only 4 participants received intensive phase treatment with 2RHZE, and 1 patient, with previously treated smear-positive pulmonary TB was prescribed intensive phase treatment with 2 months of RHZES and 1 month RHZE followed by 5RHE. Inpatient treatment was well tolerated with no reported rash, nausea, vomiting, optic neuritis, ototoxicity, or renal failure. Only one case of transient hepatotoxicity was reported, which did not require change of treatment regimen. There was no in-hospital mortality, and clinical status upon discharge from the hospital was improved in 96, (93%), no change in 4, (4%), or worse in 3 (3%). All children were discharged to home, with no transfers to the intensive care unit. Those clinically worse at discharge were all infants ≤ 4 months old at admission and were all diagnosed with culture confirmed Mtb. One infant was HIV-infected, had isoniazid-resistant Mtb, and was treated with 2RHZE and 4RH, and another infant had miliary TB. Of those with no change in clinical status at discharge all were under 5 years old, two were diagnosed with TB meningitis, one with osteoarticular TB, and one with intrathoracic TB. Infants < 12 months old had 11.3 times the odds of no improvement at discharge compared with children age 1–15 (OR 11.3, 95%CI 2.0―62.2, p = 0.006) and those with severe TB had 4.4 times the odds of no improvement compared with those with other sites of TB infection (OR 4.4, 95%CI 0.89–21.8, p = 0.07).

## Discussion

There are few studies characterizing the demographics, clinical presentation, radiographic and microbiologic findings, and treatment among children diagnosed with TB disease in Northern Viet Nam. Extrathoracic TB and severe TB were remarkably common in our study, culture confirmation rates were low, treatment well tolerated, and outcomes favorable. Half of children with TB were under 5, a consistent finding in population-based studies [4], [6], [13] and highlighting the need for optimal TB prevention, diagnosis, and treatment in this vulnerable age group. In our study, 27% of children were exposed to a known adult with TB disease, prior studies finding positive source cases in 25–50% [18]–[20]. Additionally, a recent contact investigation study performed in Ha Noi found on average 2.6 household contacts (16.9% were children 0–15 years old) for every case of smear positive TB [21].These findings suggest that contact investigations could play a powerful role in TB control in Viet Nam.

Extrathoracic and extrapulmonary TB were more common among our study participants (52% with extrathoracic TB and 70% with extrapulmonary TB) than typically reported elsewhere (9–39%) [6], [18], [19], [22]–[27]. For example, among children age 0–14 with TB disease reported to TB registries in Viet Nam and Cambodia, 141 of 360 (39%) were diagnosed with extrapulmonary TB [26]. It is possible that extrathoracic cases were preferentially referred to our hospital instead of diagnosed and managed locally. HIV-infection has a well-established association with increased extrathoracic TB [20], [28], but was not likely driving the high prevalence of extrathoracic TB in our cohort. During the study period, HIV testing was not uniformly performed, and we unfortunately only have HIV results from 27 subjects, revealing a 9% occurrence of HIV in our cohort, considerably higher than the national prevalence of HIV estimated at 0.29% for children and adults in 2010 [29], and comparable to an estimated 8.2% prevalence of HIV in adults with TB in Viet Nam [30]. The true HIV prevalence in our cohort was probably not remarkably high, suggested by the absence of hospital mortality as HIV and TB coinfection are typically associated with high hospital mortality [20]. More than one-third of patients were malnourished, and malnutrition was associated with increased extrathoracic TB as seen in other studies [31]–[33]. In this regard, the direction of causality is difficult to disentangle: malnutrition may increase risk for TB, but TB disease also promotes malnutrition. Regional Mtb strains, host genetic profiles, and environmental factors could also be contributory. As with other studies, we found lymphadenitis to be the most common extrathoracic manifestation [6], [23], [25], [27], [28], [34], [35], and most patients with TB meningitis presented with severe neurologic impairment [27]. Cavitary disease was uncommon, consistent with other studies of children and young adolescents [6], suggesting that our cohort of 0–15 year-olds had largely not shifted to an adult TB phenotype. As expected, severe TB was significantly more common in children under 3 years of age. Chest pain was only identified in 5–15 year-olds, likely reflective of the significantly higher proportion of pleural involvement in this age group and the difficulty localizing pain in younger children. Fever and/or failure to thrive were present in the majority of patients regardless of site of infection, and the majority of those with intrathoracic TB had cough, consistent with other studies [18]–[20], [36]. However, these findings may overestimate the true prevalence of these symptoms in childhood tuberculosis, as fever, failure to thrive, and cough were used to help establish a clinical diagnosis of TB, therefore less symptomatic children with TB may have been misclassified as not having TB. It is essential to have a high index of suspicion for diagnosing TB in children from high prevalence areas such as Viet Nam [15].

Establishing an accurate diagnosis in children with suspected TB can be challenging. Culture confirmation rates were low in our study, consistent with most other studies which report culture confirmation rates between 10 and 40% [2], [6]–[12]. For instance, a recent diagnostic study at the National Hospital of Pediatrics in Ha Noi found a smear positivity of 8.8% and culture positivity of 38.9% among 113 children clinically diagnosed with TB [37]. Few studies have reported higher culture confirmation rates, up to 62% [18], [19], [38]. Mtb culture confirmation rates are much lower for children than for adults. For instance, among patients diagnosed with TB disease who underwent Mtb culture testing in the United States between 1993 and 2001, 29% of children < 15 years old were culture positive compared with 79% of adults. Interestingly, culture confirmation rates were significantly higher in infants compared with older children, a finding seen in other studies [39]. Healthcare providers often hesitate in obtaining specimens for culture from infants with suspected TB. On the contrary, our results suggest that infants are an exceedingly important age group from which to obtain specimens for culture, because morbidity associated with missing the diagnosis of TB in infants is high, and because culture sensitivity appears to be higher in this age group. Smear positivity was surprisingly high (18%) compared with culture positivity (21%), likely in part due to false positive smears. Four specimens were initially read as smear positive but later found to be culture negative. Interestingly, 2 of these were fecal specimens, a potentially more difficult source to perform accurate Ziehl-Neelsen smear microscopy on.

The TST is typically positive in intrathoracic and non-severe extrathoracic TB (80–90%) [6], [18]–[20] but considerably less sensitive for TB disease in some studies (50–60%) [10], [40]. The range of sensitivities is wide and likely dependent on several factors. Malnutrition, evident in more than 1/3^rd^ of our study participants and associated with false negative TSTs, likely contributed to the low TST positivity (51%) seen in our study. Examining the association between positive TST and clinically diagnosed TB is inherently biased, since a positive TST is widely used as a clinical criterion for diagnosing TB disease, as it was in this study, resulting in considerable variability from study to study depending on how much weight is placed on TST in the clinical diagnostic algorithm.

Treatment was well tolerated with only one report of transient hepatotoxicity resolving without change in medication. Standard multidrug TB regimens are remarkably well tolerated among pediatric patients, with adverse drug reactions necessitating modification of chemotherapy noted in only 1–3% of children [39], [41]–[43]. In a recent systematic review, Donald reported rates of elevated hepatic transaminases consistently lower in children on multidrug intensive phase therapy (8–10%) than adults (11–35%), with clinically evident drug induced hepatotoxicity in only 0.22–1.3% of children [44]. Remarkably, no nephrotoxicity or neurotoxicity was noted with streptomycin use despite its inclusion in 66% of intensive phase regimens.

Clinical outcomes were excellent, with 93% clinically improved upon discharge and no in-hospital mortality. Clinical outcomes are usually better in children with TB than adults, with mortality typically ranging between 0 and 2.2% [6], [18], [19], [39] but as high as 6.9% in high HIV burden settings [20], and 17% in settings with poor treatment completion rates [23]. There are several possible explanations for the impressive outcomes evident in our study. Hospital stays were long allowing patients to complete a substantial portion of intensive phase treatment with optimal adherence under close monitoring. Drug resistance rates appeared to be low, perhaps affording better responses to therapy. It is possible that some children with benign self-resolving conditions were misclassified as having probable TB, falsely decreasing mortality. This is unlikely as children referred to NLH were in general quite ill with conditions that would worsen without appropriate therapy. Even though 10/12 (83%) of patients with TB meningitis were clinically improved at discharge, there were likely persistent neurologic sequelae in some of these patients, and this discharge data was not abstracted. As seen in other studies, infants and children with severe TB had poorer outcomes [18], [35], [39].

Our study was limited by a small sample size and hospital based study design. NLH is the only nationally designated pediatric TB referral hospital in Northern Viet Nam. As such, our study sample may not be representative of pediatric inpatient TB experiences at other health centers in Northern Viet Nam. However, this referral hospital setting afforded us access to high quality microbiologic, histopathologic, radiographic, and invasive testing not uniformly available in other Northern Viet Nam settings. The study data collected is from 2007. It is important to realize what modifications have occurred in TB management at NLH since then in order to assess the generalizability of our results to current settings. National protocols for referrals to NLH, duration of therapy at NLH, and patient discharge have remained largely unchanged. Clinical and microbiologic evaluation remain unchanged. Although the rapid molecular diagnostic system GeneXpert is now available at NLH, it is not routinely utilized due to testing expense. HIV testing in children with TB at NLH, once infrequently performed, has now become routine. There has also been a shift away from streptomycin, with greater use of ethambutol.

We describe the demographics, clinical characteristics, microbiologic and radiographic findings, treatment and outcomes among children admitted to a national referral hospital in Northern Viet Nam. Half of our study participants were less than 5 years old, and malnutrition was common. Extrathoracic TB was diagnosed in the majority of patients, and TB meningitis presented with severe neurologic impairment. Culture confirmation rates were low, treatment well tolerated despite the use of streptomycin, and outcomes favorable with no in-hospital mortality. Further studies are needed to better prevent, diagnose and treat childhood TB in Viet Nam.

## Supporting Information

Form S1 Standardized Chart Abstraction Form.
(PDF)
