# Supplementary material for: Childhood Tuberculosis in Northern Viet Nam: A Review of 103 Cases
Source: PLoS One. 2014 May 12;9(5):e97267. doi: 10.1371/journal.pone.0097267 (PMC4018290; doi:10.1371/journal.pone.0097267)
Supplement: Form S1 — Standardized Chart Abstraction Form. (PDF) [file pone.0097267.s001.pdf]

DEMOGRAPHICS

1) Patient Study Code:
☐☐☐

2) Patient Hospital Medical Record Number:
☐☐☐☐

3) Patient Name: ______________________

4) Date of birth
☐☐ / ☐☐ / ☐☐

5) Gender: ☐ Male
☐ Female

6) Ethnicity: ☐ Kinh
☐ Other (specify)_____________

7) Nationality: ☐ Vietnam
☐ Other (specify)_____________

8) Patient Address:
Number/Street _________________
Commune _____________________
District _______________________
City _______________________

9) Referred from
☐ Home/Family
☐ District Hospital
☐ City Hospital
a) Date of admission to local hospital (if applicable)
☐☐ / ☐☐ / ☐☐

10) Date of admission to National Hospital
☐☐ / ☐☐ / ☐☐

11) Date of discharge from National Hospital
☐☐ / ☐☐ / ☐☐

12) Discharged to
☐Deceased
☐Home/Family
☐District Hospital → **see q.12a**
☐City Hospital → **see q.12a**

a) **If transferred to other hospital**, list hospital address:
Number/Street _________________
Commune _____________________
District _______________________

CLINICAL CHARACTERISTIC

13) Sites of TB (mark all applicable)

☐ Pulmonary → **answer 15a**
☐ Pleural → **answer 15b**
☐ Lymphatic - Extrathoracic → **answer 15c**
☐ Meningeal → **answer 15d**
☐ Osteoarticular → **answer 15e**
Specify: ☐ Skeletal
☐ Long bone
☐ Joint
☐ Other
☐ Abdominal → **answer 15f**
Specify: ☐ Peritoneal
☐ Intestinal
☐ Other
☐ Miliary/ Disseminated
☐ Other (specify)___________
☐ Unknown

14) Systemic Symptoms	Duration (Leave blank if unknown)
☐ Fever	______days
☐ Fatigue	______days
☐ Weight-loss or failure to gain weight appropriately	______days

15) Focal Symptoms:		Duration (Leave blank if unknown)
a) Pulmonary TB	☐ Cough	______days
	☐ Hemoptysis	______days
	☐ Dyspnea	______days
	☐ Other	______days
b) Pleural TB	☐ Cough	______days
	☐ Chest pain	______days
	☐ Dyspnea	______days
	☐ Other	______days
c) TB Meningitis	☐ Headache	______days
	☐ Nausea	______days
	☐ Seizure	______days
	☐ Drowsiness, disorientation	______days
	☐ Cranial nerve palsies	______days
	☐ Coma	______days
	☐ Hemiplegia/Quadriplegia	______days
	☐ Other	______days
d) TB Lymphadenitis	☐ Lymphadenopathy	______days
e) Osteoarticular TB	☐ Localized pain	______days
	☐ Kyphosis	______days
	☐ Altered gait	______days
	☐ Other	______days
f) Abdominal TB	☐ Abdominal Pain	______days
	☐ Nausea/Vomiting	______days
	☐ Diarrhea	______days
	☐ Other	______days

16) Prematurity:
☐Y ☐N

17) Birth Weight:
☐☐ . ☐ kg

18) Significant prior medical problems
☐Y ☐N
If yes, specify ___________________

19) History of TB contact
☐Y ☐N

20) Admission Height:
☐☐☐ cm

21) Admission Weight:
☐☐ . ☐ Kg

22) Has the patient had previous history of TB prior to admission to National Hospital?
☐Y ☐N

a) **If yes**, what were sites of disease?
- ☐ Pulmonary
- ☐ Pleural
- ☐ Lymphatic - Extrathoracic
- ☐ Meningeal
- ☐ Osteoarticular
  - Specify: ☐ Skeletal
  - ☐ Long bone
  - ☐ Joint
  - ☐ Other
- ☐ Abdominal
  - Specify: ☐ Peritoneal
  - ☐ Intestinal
  - ☐ Other
- ☐ Miliary/ Disseminated
- ☐ Other (specify)___________
- ☐ Unknown

b) **If yes**, was patient treated? ☐Y ☐N ☐Unknown

1. **If yes**, list regimen:
   a) Intensive phase
   - ☐ 2SHRZ
   - ☐ 2SHRZE/1HRZE
   - ☐ 2SHRZE
   - ☐ 2HRZ
   - ☐ Other (specify)___________

   b) Continuation phase
   - ☐ 6HE
   - ☐ 4HR
   - ☐ 5R3H3E3 or 5HRE

☐ Other (specify)___________

2. **If yes**, what is patient's treatment status prior to admission to National Hospital?
   ☐Completed (treatment completion)
   ☐Cured (treatment completion and sputum microscopy negative for AFB at the end of treatment and one previous occasion)
   ☐In progress
   If in progress, how many days of therapy has patient received? ____ days
   ☐Defaulted (treatment interrupted for 2 or more months)
   ☐Unknown
   ☐Other (specify)___________

23) Tuberculin Skin Test (Mantoux) result:
☐☐ mm

24) BCG Vaccination
☐Y ☐No/Unknown

25) HIV Status
☐Positive ☐Negative ☐Unknown

Diagnostics

26) Chest Radiograph Results
☐Normal ☐Abnormal

a) **If abnormal**, what is pattern of abnormality? (mark all applicable)
   ☐Lobar consolidation
   ☐Cavitary lesion
   ☐Micronodular lesions
   ☐Intrathoracic adenopathy
   ☐Miliary
   ☐Pleural effusion
   ☐Other abnormalities

27) Biopsy result: (if not performed, leave blank)

a) Location:
   ☐Bone
   ☐Lymph node
   ☐Skin
   ☐Liver
   ☐Bone marrow
   ☐Other (specify)_________

b) Result:
   ☐Typical tuberculosis (caseating epithelioid granuloma)
   ☐Atypical features but suggests tuberculosis (noncaseating granuloma)
   ☐Other

28) Culture and microscopy results:

Date Specimen Obtained:	Source:	AFB Smear:	M.tb Culture:
☐☐ / ☐☐ / ☐☐	☐☐		

__ __ / __ __ / __ __	__ __	☐+ ☐-	☐+ ☐-
__ __ / __ __ / __ __	__ __	☐+ ☐-	☐+ ☐-
__ __ / __ __ / __ __	__ __	☐+ ☐-	☐+ ☐-
__ __ / __ __ / __ __	__ __	☐+ ☐-	☐+ ☐-
		☐+ ☐-	☐+ ☐-

Source code:

01 sputum	*04 Pleural fluid*	*07 Urine*
02 gastric lavage	*05 CSF*	*08 Other biopsy (specify)___________*
03 bronchoscopy washing	*06 LN biopsy*	*09 Other fluid (specify)__________*

a) **If culture is positive**, indicate resistance pattern:

	**Sensitive**	**Resistant**
Rifampin	☐	☐
INH	☐	☐
PZA	☐	☐
EMB	☐	☐
Strep	☐	☐

Treatment Regimen

29) Prescribed Regimen

a) Planned regimen:

1) Intensive phase

☐ 2RHZS

☐ 2RHZES/1RHZE

☐ 2RHZES

☐ 2RHZ

☐ Other (specify)___________

2) Continuation phase

☐ 6HE

☐ 4RH

☐ 5RHE

☐ Other (specify)____________

b) Documented duration **at National Hospital** _____ days

30) Was treatment interrupted during this hospitalization?

☐Y ☐N

a) **If yes**, what was reason for interruption? _______________________

31) Were there adverse events to therapy requiring discontinuation?

☐Y ☐N → **skip to q.33**

a) **If yes,** after how many days? _______days

b) **If yes**, what was adverse reaction?

☐hepatotoxicity

☐rash

☐nausea/vomiting

☐arthritis

☐optic neuritis

☐other (specify)___________

c) **If yes**, to what drug?
☐INH
☐Rifampin
☐PZA
☐Other (specify)___________

d) **If yes,** what is alternate regimen used? ____________

e) **If yes,** documented duration of alternative regimen at National Hospital ____ days

32) Were there adverse events to alternative regimen requiring discontinuation?
☐Y ☐N → **skip to q.33**

f) **If yes,** after how many days? ______days

g) **If yes**, what was adverse reaction?
☐hepatotoxicity
☐rash
☐nausea/vomiting
☐arthritis
☐optic neuritis
☐other (specify)___________

h) **If yes**, to what drug?
☐INH
☐Rifampin
☐PZA
☐Other (specify)___________

i) **If yes,** what is alternate regimen used? ____________

j) **If yes,** documented duration at National Hospital ____ days

33) Were steroids used?
☐Y ☐N → **skip to q.34**

a) **If yes,** which drug
☐Prednisone
☐Methylprednisolone
☐Other

b) **If yes,** list dose ______mg

c) **If yes,** planned duration _____ days

d) **If yes,** documented duration at National Hospital ______ days

34) Were any **THERAPEUTIC** surgical intervention performed?
☐Y ☐N

If yes, please specify procedure:
____
____

Date:
☐☐ / ☐☐ / ☐☐

OUTCOME

If deceased, disregard this section.

35) Discharge Weight:

☐☐ . ☐ kg

36) Discharge Height:

☐☐☐ cm

37) Documented status at time of discharge

☐Resolved ☐Improved ☐No change ☐Worse ☐Unavailable → **see q.37a**

a) **If documented status is unavailable**, what is status of systemic signs and focal symptoms (**q.14 and q.15**) at time of discharge based on progress notes at time of discharge:

☐Improved ☐No change ☐Worse ☐Unable to assess
